# Deamidated Zein Peptide Nanoparticles for Enhanced Quercetin Delivery: Structural Analysis, Stability, and Antioxidant Properties

**DOI:** 10.3390/gels12060506

**Published:** 2026-06-07

**Authors:** Ying Kuang, Ting Zhang, Hui-Yu Liu, Jia-Peng Wu, Wen Luo, Kai Chen, Hong Qian, Kao Wu, Cao Li

**Affiliations:** 1 Key Laboratory of Fermentation Engineering (Ministry of Education), Cooperative Innovation Center of Industrial Fermentation (Ministry of Education & Hubei Province), National “111” Center for Cellular Regulation and Molecular Pharmaceutics, Glyn O. Phillips Hydrocolloid Research Centre at HBUT, School of Life and Health Sciences, Hubei University of Technology, Wuhan 430068, China; kuangying@hbut.edu.cn (Y.K.); licao@hbut.edu.cn (C.L.); 2 School of Nursing and Health Management, Wuhan Donghu University, Wuhan 430212, China

**Keywords:** deamidated zein peptide, quercetin, nanoparticles, delivery system, stability

## Abstract

To address the poor solubility, instability, and low oral bioavailability of quercetin (Q), Q-loaded nanoparticles (Q@DDZ) were fabricated using deamidated zein peptide (DDZ) via a pH-driven method. As a food-grade hydrophilic colloid, DDZ effectively improves the colloidal stability of the delivery system. Deamidation increased hydrophilic amino acids and surface negative charge. DDZ bound Q via static quenching with a higher binding constant (K_a_ = 2.25 × 10^3^ L/mol) and more binding sites (*n* = 1.7561) than zein, along with stronger hydrogen bonding and hydrophobic interactions. Q@DDZ exhibited higher encapsulation efficiency (45.36–87.32%) and loading capacity (1.82–12.27%) than Q@zein, with a smaller particle size and better dispersibility. At 50.0 μg/mL Q, Q@DDZ showed 41.06% (DPPH) and 46.62% (ABTS) higher scavenging rates than free Q. It displayed excellent stability under acidic, high ionic strength, and thermal conditions (80 °C, 180 min). In simulated digestion, Q@DDZ delayed Q release in the oral and gastric phases and prolonged intestinal release, which indicated potentially improved bioavailability. This study provides mechanistic insights into deamidation-modified plant protein delivery systems for hydrophobic bioactives, offering new perspectives for the development of functional biopolymer gel materials.

## 1. Introduction

Quercetin (Q), a natural flavonoid widely distributed in fruits, vegetables, grains, and herbal medicines, has been proven to possess physiological effects, including antioxidant [1], antitumor [2], lipid-lowering [3], cardiovascular [4], and antiglycation [5] activities. However, its practical application in functional foods and pharmaceuticals is severely limited by its extremely poor aqueous solubility, low oral bioavailability [6], and susceptibility to degradation under environmental stresses such as light, temperature, and pH levels [7].

To overcome these challenges, encapsulation within nanoscale delivery systems based on food-grade biopolymer colloids and food-derived hydrophilic gel matrices has emerged as an effective strategy for enhancing the stability, solubility, and controlled release of hydrophobic bioactive compounds [8]. Distinct from synthetic carriers, natural protein-based colloidal delivery systems possess unique advantages in food applications, including excellent biocompatibility, inherent food safety, and significant potential for bioactive component protection and undesirable flavor mitigation, which makes them ideal carriers for hydrophobic nutraceuticals. Among various carriers, food-derived proteins have attracted considerable attention due to their biocompatibility, biodegradability, and functional versatility [9]. Zein, a major prolamin found in corn, provides substantial benefits as a natural protein carrier [10]. Its low allergenicity has been demonstrated to mitigate safety risks in applications, and its high content of hydrophobic amino acids facilitates strong interactions with hydrophobic compounds such as Q, which is critical for stabilizing colloidal particle assemblies [11,12,13]. As demonstrated in previous studies, zein-based nanoparticles have been shown to improve the encapsulation efficiency and antioxidant stability of Q [14]. Nevertheless, the weak colloidal stability and limited hydrophilicity of native zein restrict the performance of such protein colloid delivery systems, and zein’s inherent hydrophobicity and tendency to aggregate near its isoelectric point (pI ≈ 6.2) limit the delivery efficacy and stability of zein-based delivery systems [15].

Protein modification techniques, including glycosylation, enzymatic modification, and deamidation [16,17], offer a viable approach to optimize the physicochemical properties of zein for enhanced delivery performance. While glycosylation has been demonstrated to improve protein solubility, thermal stability, and dispersibility, typically through Maillard conjugation with polysaccharides [18,19], it frequently introduces uncontrollable browning and complex reaction by-products [20,21]. Enzymatic modification improves the water solubility of protein and bio-accessibility by cleaving peptide bonds [22], yet it may reduce molecular weight and structural integrity with excessive hydrolysis, thereby generating bitter peptides. Deamidation is a process involving the hydrolysis of glutamine (Gln) and asparagine (Asn) residues to aspartic (Asp) and glutamic (Glu) under mild conditions without introducing foreign molecules [23]. This process simultaneously increases a protein’s hydrophilicity and net negative charge [24], reduces its allergenic potential by disrupting conformational epitopes [25], and introduces carboxyl groups that enhance the potential for hydrogen bonding and electrostatic interactions with the phenolic hydroxyls of Q [26]. These advantages make deamidation particularly suitable for developing functional zein-based colloidal nanoparticle dispersion systems with improved colloidal stability.

In this study, Q-loaded nanoparticle systems were constructed using deamidated zein peptide (DDZ) via a pH-driven method. The structural alterations in zein induced by deamidation were systematically characterized to clarify the impact of deamidation modification on gel formation potential. The interaction mechanisms between the carrier and Q were investigated, along with the self-assembly behavior of the nanoparticles. On this basis, the key performance attributes of the two delivery systems were compared, including encapsulation efficiency, antioxidant activity retention, colloidal stability, and in vitro release behavior. This work provides a promising strategy to improve Q oral bioavailability and offers theoretical insights for designing plant protein-based delivery systems for lipophilic nutraceuticals.

## 2. Results and Discussion

### 2.1. Structural Characterization of DDZ

The hydrolysis and deamidation degrees of DDZ are presented in Table 1. These results indicated that the modification process caused only mild and limited cleavage of the zein main chain, without triggering excessive degradation or molecular fragmentation. The conversion of side-chain amides to carboxyl groups increased the protein’s surface negative charge and hydrophilicity, while retaining sufficient hydrophobic regions, thereby enabling the efficient encapsulation of hydrophobic active substances.

**Figure 1:**
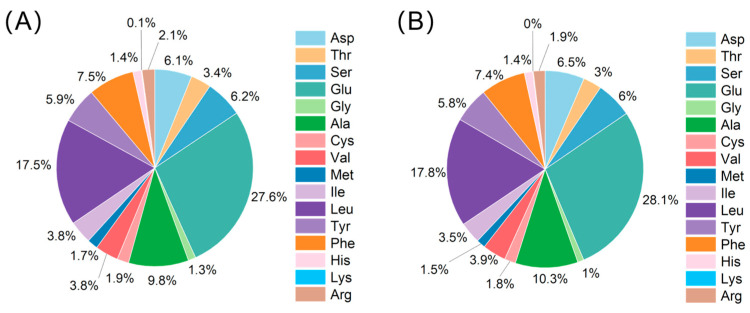
Amino acid composition and content of zein (**A**) and DDZ (**B**).

**Figure 2:**
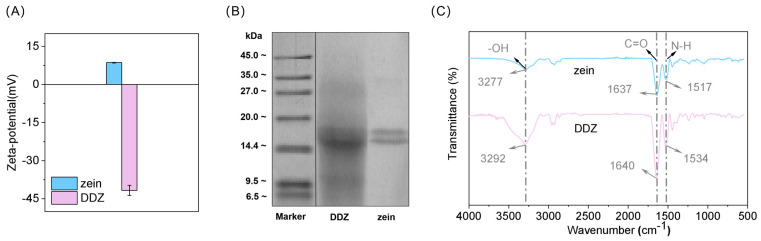
Zeta potential (**A**), molecular weight distribution (**B**), and FTIR spectra (**C**) of zein and DDZ.

Deamidation mainly involved Asn and Gln residues, which were converted to Asp and Glu, respectively. Figure 1A and Figure 1B show the amino acid composition of zein and DDZ, respectively. After deamidation, the proportion of Asp increased from 6.11% to 6.46%, and that of Glu increased from 27.56% to 28.13%. This indicated that deamidation hydrolyzed the amide groups (-CONH_2_) to generate corresponding carboxyl groups (-COOH), thereby increasing the contents of hydrophilic amino acids like Asp and Glu, and resulting in the decrease in the protein’s hydrophobicity and the increase in its polarity, which is essential for stable colloidal nanoparticle dispersions. This result was consistent with the zeta-potential measurements shown in Figure 2A. Zein exhibited a weak positive surface potential, whereas the DDZ-treated sample showed a significantly more negative potential. The marked increase in negative surface charge was directly attributed to carboxyl groups newly generated during deamidation, which enhanced electrostatic repulsion between protein molecules. The increased polarity and surface charge promoted the formation of a stable colloidal dispersion, as stronger electrostatic repulsion inhibited aggregation and improved system stability.

The molecular weight distribution of zein and DDZ is shown in Figure 2B. Native zein exhibited distinct bands within the 14.4–20.0 kDa range with a concentrated, uniform molecular weight distribution. In contrast, the DDZ sample exhibited a diffuse, broad band pattern centered predominantly around 14.4 kDa with no obvious single molecular weight band, indicating a shift towards lower molecular weights. These results suggested that the zein proteins underwent significant degradation or structural depolymerisation during deamidation treatment, resulting in a broader molecular weight distribution and a pronounced reduction in the average molecular weight. Deamidation modification directly alters the surface properties and spatial conformation of the zein molecule, which may affect its binding interaction with Q.

The Fourier transform infrared spectra of zein and DDZ are shown in Figure 2C. Compared with zein, the absorption band of DDZ in the 3300–3500 cm^−1^ region shifted to higher wavenumbers, with increased intensity and broadening. This was attributed to the overlap of N–H stretching and the newly formed O–H stretching of carboxyl groups, confirming carboxyl generation and enhanced hydrogen bonding upon deamidation. In the amide I region (1600–1700 cm^−1^), shifts in the characteristic C=O peak were observed, likely resulting from the conversion of side-chain amide groups to carboxyl groups. In the amide II region, the N–H peak exhibited a blue shift and increased intensity, indicating that deamidation caused amide bond cleavage and structural modification of the protein. Quantitative analysis of the ratio of the peak areas of the carboxyl and amino groups further corroborated the above conclusion. Calculations showed that this ratio was 0.62 for natural corn protein, whereas the ratio for DDZ was 0.43. The decrease in this ratio fully reflected the consumption of amide groups and the formation of carboxyl groups during the deamidation reaction, providing additional quantitative evidence of the success of the protein modification.

### 2.2. Molecular Interaction Analysis

**Figure 3:**
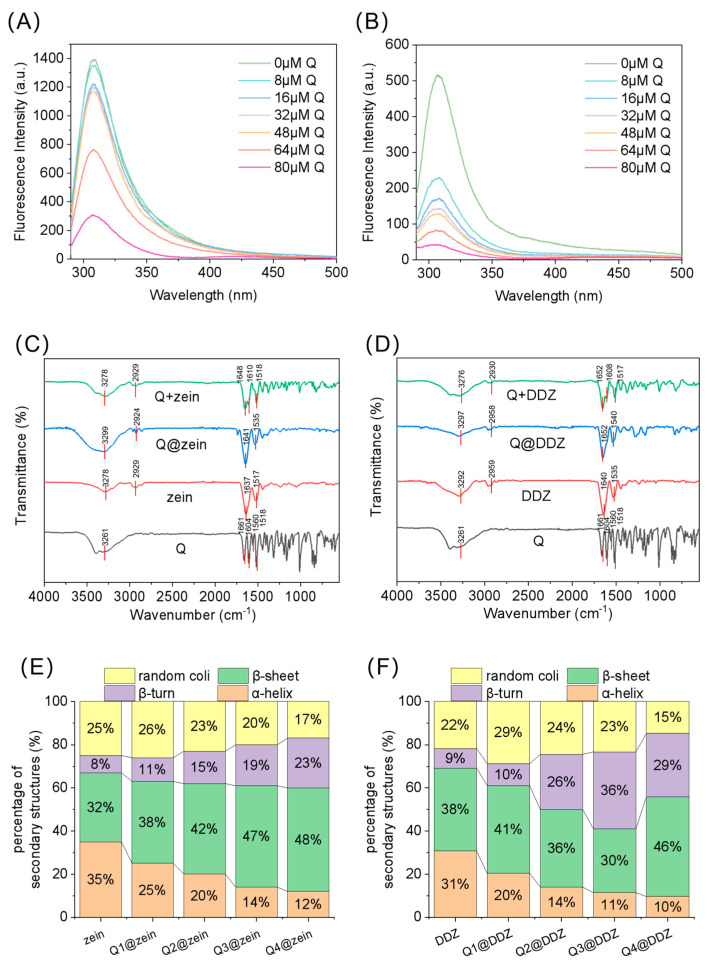
Intrinsic fluorescence emission spectra of zein (**A**) and DDZ (**B**) with increasing Q concentrations (0.0–80.0 μmol/L). FTIR spectra of zein, Q, their physical mixtures, and Q@zein (**C**) and of DDZ, Q, their physical mixtures, and Q@DDZ (**D**). Secondary structure composition of zein and Q@zein (**E**) and DDZ and Q@DDZ (**F**) at different Q loading levels.

Under an excitation wavelength of 280 nm, the intrinsic fluorescence of zein mainly originates from tyrosine (Tyr) residues. As shown in Figure 3A,B, the λ_max_ value did not change in the presence of Q, but the Tyr fluorescence intensity decreased with increasing Q concentration. Thus, the Tyr fluorescence was quenched by Q. The data in Figure 3A,B were analyzed using the Stern–Volmer equation, and the relevant quenching rate constants were calculated to investigate the fluorescence quenching mechanism. The quenching rate constant is critical for determining the dynamic and static quenching behavior between molecules. As shown in Table 2, the molecular quenching rate constants (K_q_) of both zein and DDZ were significantly higher than the maximum dynamic quenching constant (2 × 10^10^ M^−1^ s^−1^), indicating that the Tyr fluorescence quenching of zein and DDZ was caused by the specific interaction between the quencher (Q) and the protein, following a static quenching mechanism.

The binding affinity of Q to the protein and the number of available binding sites were represented by the binding constant (K_a_) and the number of binding sites (*n*) in Table 2, respectively. Compared with zein, DDZ had higher K_a_ and n, suggesting that DDZ has superior potential for constructing robust colloidal gel carriers for hydrophobic compounds.

The results of the FTIR analysis demonstrated the presence of distinct intermolecular interactions between Q, zein, and DDZ. As demonstrated in Figure 3C,D, the free Q compound exhibited phenolic hydroxyl (-OH) stretching vibration peaks at 3261 cm^−1^ and 1661 cm^−1^, aromatic ring C=O stretching vibration peaks at 1661 cm^−1^ and 1604 cm^−1^, and aromatic ring skeletal vibration peaks at 1560 cm^−1^ and 1518 cm^−1^, which were clearly retained in both the physical mixtures of zein with Q and DDZ with Q. Conversely, these peaks were completely absent in Q@zein and Q@DDZ, thereby directly confirming the successful embedding of Q within the carrier. In the Q@zein system, a shift in the amide I band representing C=O stretching to 1641 cm^−1^ was observed, accompanied by a shift in the amide II band representing N-H bending and C-N stretching to 1535 cm^−1^. These observations suggested the presence of stronger hydrophobic interactions between zein’s amide groups and Q’s benzene rings. Concurrently, the -OH peak shifted to 3299 cm^−1^, indicative of the formation of new hydrogen bonds between zein’s hydroxyl groups and Q’s phenolic hydroxyl groups. For Q@DDZ, the amide I band shifted to 1652 cm^−1^, and the amide II band shifted to 1540 cm^−1^, with greater shifts than in Q@zein, indicating stronger hydrophobic interactions between DDZ and Q. The -OH peak shifted to 3297 cm^−1^, with a similarly more pronounced shift, reflecting more stable intermolecular bonding within the colloidal dispersion system.

As demonstrated in Figure 3E,F, the alterations in secondary structure content of zein, DDZ, Q@zein and Q@DDZ at different Q loading levels were evident. Zein exhibited 35% α-helix, 32% β-sheet, 8% β-turn, and 25% random coil. DDZ exhibited a 31% α-helix configuration, 38% β-sheet conformation, 9% β-turn structure, and 22% random coil composition. This finding suggested that the carboxyl group, introduced by deamidation, disrupted the original hydrogen bond network within the zein molecule, resulting in the partial unwinding of the α-helix structure. Subsequent to this, the peptide segments underwent a process of rearrangement through hydrophobic interactions and the formation of new hydrogen bonds, resulting in the establishment of a more stable β-sheet structure. The proportions of β-turns and random coils increased marginally, enhancing the flexibility of the deamidated protein molecules and providing a greater number of conformational possibilities for subsequent binding with Q. The incorporation of varying concentrations of Q was demonstrated to result in corresponding alterations to the composition of the protein’s secondary structure. As the content of Q increased, α-helix structures underwent a progressive process of denaturation, resulting in the relative loosening of the protein’s internal spatial structure and the creation of greater capacity for Q to embed within it. The secondary structure of the protein shifted towards β-folding and β-turns, facilitating the formation of hydrophobic cavities that encapsulate Q.

### 2.3. Morphological Characterization

**Figure 4:**
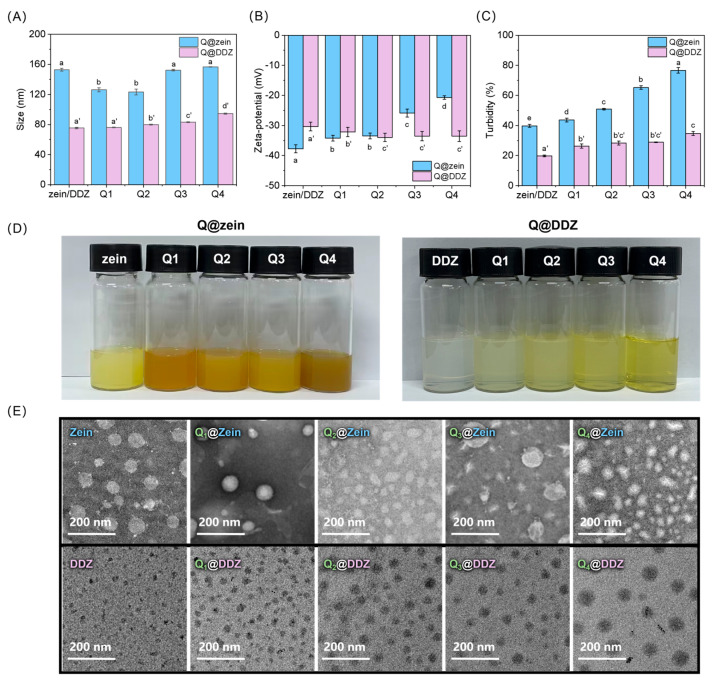
Particle size distribution (**A**), zeta potential (**B**), turbidity (**C**), appearance (**D**), and TEM images (**E**) of Q@zein and Q@DDZ (Q1, Q2, Q3, and Q4 represent carrier protein-to-Q loading ratios of 40:1, 20:1, 10:1, and 5:1, respectively). Different letters mean values showed significant difference (*p* < 0.05).

The particle size distribution and zeta potential can effectively evaluate the characteristics of nanoparticles. Figure 4A,B show the particle size distribution and zeta potentials of Q@zein and Q@DDZ. As the Q load increases, both Q@zein and Q@DDZ particle sizes demonstrated an upward trend; however, the Q@DDZ particle size remained smaller than the Q@zein particle size. The zeta potentials of all particles were negative, indicating that the nanoparticle surfaces carried abundant negative charges; the absolute value of the zeta potential of Q@DDZ was consistently higher than that of Q@zein.

As the feed amount increased, more Q was incorporated into the carrier protein, leading to an increase in particle size. During deamidation, Asn and Gln residues in zein were converted to Asp and Glu, introducing more carboxyl groups. These carboxyl groups increased the negative charge density on the protein molecular surface, enhanced the electrostatic repulsion between molecules, and thus prevented particle aggregation, resulting in a more robust colloidal dispersion. The smaller size and higher surface charge density of Q@DDZ enhanced dispersibility, thereby reducing colloidal instability caused by aggregation. Consequently, this improved the storage and processing stability of the colloidal nanoparticle delivery system. This structure provided a larger specific surface area and more efficient Q binding sites.

The turbidity changes in Q@zein and Q@DDZ are shown in Figure 4C. As the Q proportion increased, the turbidity of both Q@zein and Q@DDZ exhibited an upward trend. At the same Q proportion, the turbidity of Q@zein consistently remained significantly higher than that of Q@DDZ. This result was consistent with the appearance of the sample shown in Figure 4D. Zein possessed stronger hydrophobicity; upon binding with Q, Q@zein readily underwent hydrophobic aggregation, leading to increased particle size and enhanced light scattering, thereby exhibiting higher turbidity. In contrast, DDZ exhibited enhanced hydrophilicity, enabling more uniform Q binding and improved particle dispersion. This reduced aggregation tendency, resulting in lower turbidity and a more gradual increase over time.

TEM images of Q@zein and Q@DDZ are shown in Figure 4E. Self-assembled zein nanoparticles exhibit size heterogeneity, irregular morphology, and pronounced agglomeration. After loading Q, the Q@zein particles increased in size but exhibited intensified agglomeration. In contrast, DDZ particles formed significantly smaller, more uniform, and highly dispersible aggregates due to enhanced electrostatic repulsion from increased surface negative charge density, contributing to homogeneous colloidal assemblies. After Q loading, Q@DDZ particles only slightly increased in size while maintaining excellent uniformity and dispersion without noticeable agglomeration. Strong hydrophobic interactions in native zein cause particle agglomeration. However, deamidation modification increases negative surface charge, effectively inhibiting protein aggregation. Furthermore, after Q loading, enhanced electrostatic repulsion counteracts the hydrophobic attraction caused by Q embedding into the hydrophobic core. This optimized nanoparticle structure and dispersion make them more suitable as drug delivery carriers.

### 2.4. Surface Hydrophobicity Analysis

**Figure 5:**
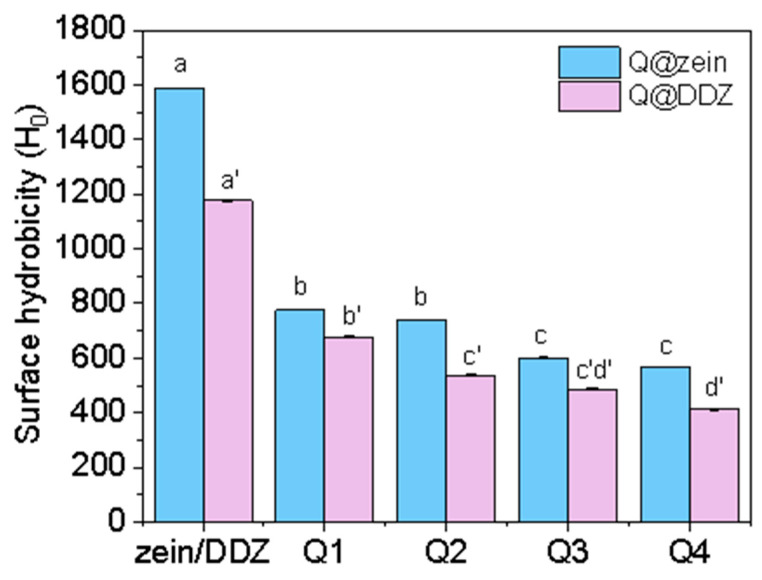
Surface hydrophobicity of Q@zein and Q@DDZ (Q1, Q2, Q3, and Q4 represent carrier protein-to-Q loading ratios of 40:1, 20:1, 10:1, and 5:1, respectively). Different letters mean values showed significant difference (*p* < 0.05).

Changes in the protein spatial structure can directly influence colloidal assembly characteristics. ANS is a hydrophobic fluorescent probe that can interact with hydrophobic regions on the protein surface, resulting in enhanced fluorescence intensity. The surface hydrophobicity of Q@zein and Q@DDZ was shown in Figure 5. The surface hydrophobicity of the protein gradually decreased with increasing Q concentration. Deamidation converted Asn and Gln in zein to hydrophilic Asp and Glu, increasing the content of hydrophilic amino acids. Meanwhile, the introduced carboxyl groups increased the negative charge on the protein molecules, causing the protein structure to unfold. Polar groups were exposed and formed a hydration layer with water molecules, reducing surface hydrophobicity. During Q encapsulation, DDZ had stronger hydrophilicity, so Q was more easily encapsulated inside DDZ, reducing its exposure on the surface. With increasing Q dosage, more Q was stably encapsulated, further reducing the surface hydrophobicity.

### 2.5. EE and LC Analysis

**Figure 6:**
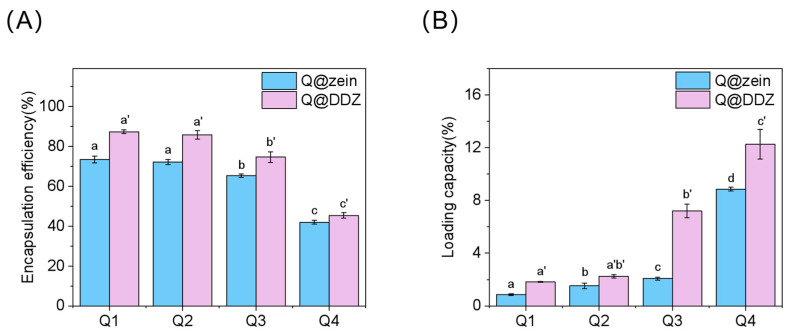
EE (**A**) and LC (**B**) of Q@zein and Q@DDZ (Q1, Q2, Q3, and Q4 represent carrier protein-to-Q loading ratios of 40:1, 20:1, 10:1, and 5:1, respectively). Different letters mean values showed significant difference (*p* < 0.05).

Figure 6A and Figure 6B show the EE and LC of Q@zein and Q@DDZ, respectively. With different Q formulations, the EE and LC of Q@DDZ were consistently higher than those of Q@zein when using the same Q formulation. With the Q2 formulation, the EE of Q@DDZ attained up to 85.75%, which was 1.19 times that of Q@zein. With the Q4 formulation, the LC reached up to 12.27%, which was 1.39 times that of Q@zein. This exceptional loading performance was attributed to the strong interaction between DDZ and Q. Calculations based on the Stern–Volmer equation suggested that, compared to zein, DDZ had a higher binding constant and more binding sites with Q, which confirmed its greater affinity for Q. According to the secondary structure analysis, deamidation loosened the protein structure, exposing hydrophobic amino acid residues (e.g., leucine (Leu), isoleucine (Ile), and phenylalanine (Phe)) that were originally encapsulated inside the protein. The hydrophobic groups of Q can form stronger hydrophobic interactions with these exposed hydrophobic amino acid residues. In addition to hydrophobic interactions, the structural changes in the protein after deamidation may also enhance hydrogen bonding. The carboxyl groups (-COOH) introduced by deamidation can act as hydrogen bond donors or acceptors, interacting with groups (-OH) in Q that can form hydrogen bonds. On the other hand, the conformational changes in the protein may expose groups inside the protein that can form hydrogen bonds, increasing the chance of hydrogen bond formation with Q. For example, amino groups (-NH_2_) or hydroxyl groups on amino acid residues can form hydrogen bonds with corresponding groups in Q after the protein structure unfolds, thereby enhancing the hydrogen bonding between DDZ and Q. For the remainder of the study, samples prepared using the Q2 loading amount were employed due to their minimal size, relatively high encapsulation efficiency, and satisfactory loading capacity.

### 2.6. Environmental Stability

**Figure 7:**
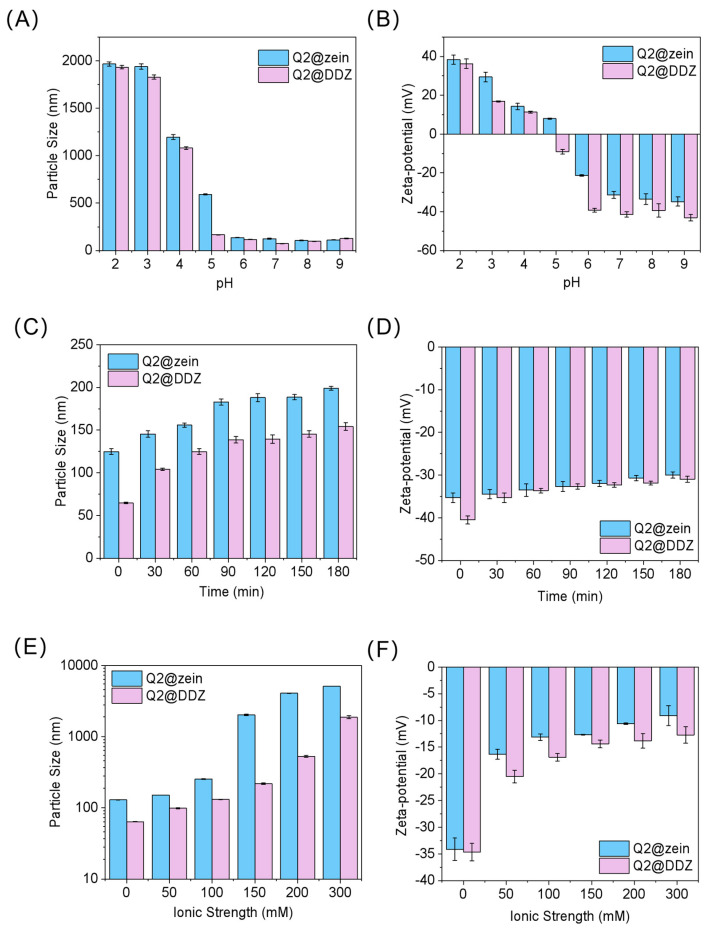
Environmental stability of Q2@zein and Q2@DDZ: pH stability (**A**,**B**), thermal stability (**C**,**D**), and ionic strength stability (**E**,**F**).

As biopolymer gel carriers, nanoparticles undergo significant changes in the gastrointestinal tract due to pH fluctuations. Therefore, pH stability is critical for their practical application. As shown in Figure 7A,B, the pI of zein is 6.2; thus, Q2@zein was prone to aggregation and destabilization between pH 5.0 and 7.0 due to weak electrostatic repulsion. After deamidation, the pI of the protein decreased; therefore, Q2@DDZ remained relatively stable in the pH range of 5.0–9.0, with a small average particle size. However, significant aggregation was observed at pH 2.0–4.0, where the zeta potential was close to 0, resulting in weak electrostatic repulsion and promoting particle aggregation.

In the near-neutral oral environment (pH 6.5–7.5), Q2@zein and Q2@DDZ exhibited good stability and dispersibility, enabling them to tightly encapsulate Q and effectively inhibit its release within the oral cavity. Upon entering the acidic gastric environment (pH 1.0–3.0), the process of H+ neutralization of the negative charge on the protein surface results in particle aggregation in both systems, accompanied by near-zero zeta potential and weakened electrostatic repulsion. Nevertheless, Q2@DDZ, with its higher negative charge density and stronger intermolecular interactions, still maintained better dispersion and slowed down the premature release of Q compared with Q2@zein. Upon entering the intestine (pH 6.0–8.0), deamidation induced increased negative charge and a more unfolded protein conformation for Q2@DDZ, thereby generating stronger electrostatic repulsion and a more stable hydrophobic cavity. This prolonged the action time of Q in the intestine, allowed sufficient contact between Q and intestinal absorption sites, improved absorption efficiency, and better supported the physiological and pharmacological effects of Q in the intestine.

Heat treatment is a common sterilization method in food processing; thus, investigating the effect of temperature on the stability of nanoparticles is of great significance. As shown in Figure 7C,D, when Q2@zein and Q2@DDZ were incubated at 80 °C for 0–180 min, the particle sizes of Q2@zein and Q2@DDZ increased gradually while their zeta potentials remained stable, indicating good thermal stability for both formulations. Deamidation modification enabled DDZ to form stronger hydrogen bonds and hydrophobic interactions with Q, thereby limiting excessive conformational changes in the protein. Consequently, Q2@DDZ consistently maintained a smaller particle size and higher negative charge density, resulting in superior thermal stability and dispersibility.

The impact of ion strength on colloidal stability necessitates investigation of its role in nanoparticle delivery systems. As shown in Figure 7E,F, with increasing ionic strength, cations shielded negative charges on the protein surface, weakening electrostatic repulsion and leading to increased particle size and reduced absolute negative zeta potential for both Q2@zein and Q2@DDZ. Deamidation modification elevated the surface negative charge density of DDZ, enabling stronger electrostatic repulsion across a range of ionic strengths. Enhanced hydrogen bonding and hydrophobic interactions with Q further stabilized the complex structure. Q2@DDZ thus consistently maintained a smaller particle size and higher electronegativity, exhibiting superior ionic strength stability. Q2@zein, with lower charge density and weaker electrostatic repulsion, was more prone to aggregation, resulting in a larger particle size increase and a more pronounced zeta-potential reduction.

### 2.7. Antioxidant Activity

**Figure 8:**
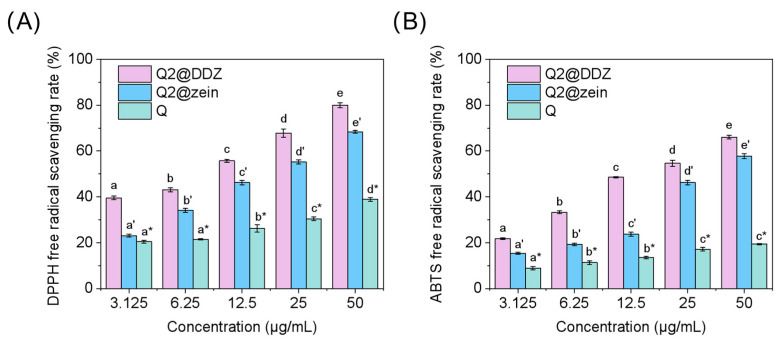
DPPH (**A**) and ABTS (**B**) radical scavenging activities of Q, Q2@zein, and Q2@DDZ. (Q stands for free quercetin. Q2 represents carrier protein-to-Q loading ratios of 20:1.) Different letters mean values showed significant difference (*p* < 0.05).

The antioxidant activity of the nanoparticles was evaluated by measuring their DPPH and ABTS radical scavenging abilities. As shown in Figure 8A,B, the radical scavenging rates of Q2@zein and Q2@DDZ increased with Q concentration and were consistently higher than those of free Q in the range of 3.125–50.0 μg/mL, while Q2@DDZ showed the highest value among the three groups. At a Q concentration of 50 μg/mL, Q2@DDZ achieved DPPH and ABTS radical scavenging rates of 79.95% and 66.02%, respectively, which were 2.06-fold and 3.40-fold higher than those of free Q, and 11.58% and 8.31% higher than those of Q2@zein. These results demonstrated that encapsulation within the protein colloidal matrix significantly preserved the antioxidant activity of Q, and deamidation further enhanced this protective effect.

Encapsulation protects Q from degradation or inactivation in the aqueous environment, thus enabling more Q to interact with and scavenge free radicals. Moreover, the unfolding of DDZ after deamidation treatment significantly improved its hydrophilicity and dispersibility in water, and optimized the structural integrity of the colloidal particles, thereby promoting the interaction between encapsulated Q and free radicals in the aqueous phase.

### 2.8. In Vitro Release Behavior

**Figure 9:**
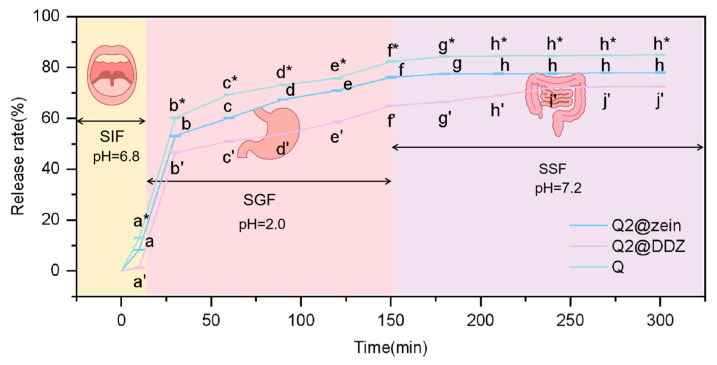
In vitro release profiles of Q, Q2@zein, and Q2@DDZ. (Q stands for free quercetin. Q2 represents carrier protein-to-Q loading ratios of 20:1.) Different letters mean values showed significant difference (*p* < 0.05).

The in vitro release behavior of Q2@zein and Q2@DDZ is shown in Figure 9. The in vitro release behavior of nanoparticles is important for evaluating their potential as oral delivery systems based on biopolymer gels. As can be seen, during the oral phase, only 1.27% of Q2@DDZ was released after 10 min, which was 10% of the amount released by free Q and 15% of the amount released by Q2@zein. This significantly reduced the premature release of Q, prevented bitterness and improved palatability. Q is prone to degradation and inactivation in acidic environments. Upon entering the highly acidic gastric phase, the release rate of Q2@DDZ after 120 min was 58.50%, which was 12.39% and 17.30% lower than the release rates of free Q and Q2@zein, respectively. This effectively delayed premature leakage of Q in the acidic environment, reducing degradation and inactivation and preserving more active ingredients for intestinal absorption. In the intestinal phase, the release peaks of free Q and Q2@zein were reached at 180 min, while the release peak of Q2@DDZ was delayed until 240 min, significantly extending the duration of sustained release in the intestine. Deamidation modification enhanced the binding affinity between DDZ and Q, enabling targeted sustained release of Q in the intestine and providing a longer window for full binding to absorption sites. This is expected to improve bioavailability and physiological efficacy.

## 3. Conclusions

This study demonstrated that deamidation is an effective strategy to optimize zein properties for the design of advanced biopolymer gels and delivery systems. Deamidation increased the hydrophilicity and negative charge density of zein by converting Asn and Gln to Asp and Glu, leading to a more unfolded structure with reduced molecular weight. DDZ exhibited higher binding affinity for Q than native zein, as evidenced by a higher binding constant (K_a_ = 2.25 × 10^3^ L/mol) and more binding sites (*n* = 1.7561). Structural analysis revealed that deamidation induced a shift in protein secondary structure from α-helix to β-sheet and β-turn, forming hydrophobic cavities for Q encapsulation. Consequently, Q@DDZ achieved higher EE (45.36–87.32%) and LC (1.82–12.27%) than those of Q@zein (EE: 41.98–73.45%; LC: 0.87–8.85%), with the Q2 formulation exhibiting an optimal balance between particle size and loading capacity. Q2@DDZ demonstrated superior colloidal stability across a broad pH range (2.0–9.0), maintained structural integrity under thermal treatment (80 °C for 180 min), and exhibited greater resistance to high ionic strength compared to Q2@zein. Furthermore, Q2@DDZ effectively preserved the antioxidant activity of Q and mitigated its premature Q release in simulated oral and gastric environments, while enabling sustained release in the intestinal phase, thereby enhancing its potential bioavailability. Collectively, these findings provide a theoretical foundation for the modification of plant proteins and the development of efficient delivery systems for bioactive compounds, thereby expanding the application prospects of DDZ in functional foods and pharmaceuticals.

## 4. Materials and Methods

### 4.1. Materials

Zein (analytical grade) was purchased from J&K Scientific (Beijing, China). The zein supplied by the vendor contains ~14.15% nitrogen, 4.85% moisture, and 0.25% ash (CAS: 9010-66-6). Q (purity ≥ 98.0%) was purchased from Macklin company (Shanghai, China). Other chemical reagents are of analytical grade.

### 4.2. Preparation of DDZ

DDZ was prepared based on previously reported methods [27], with minor modifications. Zein was dissolved in a 70% ethanol solution of 0.1 M NaOH, and the resulting mixture was left to react for 24 h at 25 °C. Ethanol was removed by rotary evaporation. The pH of the solution was adjusted to 3.0 by dropwise addition of HCl, and protein precipitated within 12 h. The precipitate was rinsed repeatedly with deionized water, dissolved in 2 M NaOH solution and adjusted to pH 9.0, and then dialyzed using dialysis bags with a molecular weight cut-off (MWCO) of 3.5 kDa. The freeze-dried product yielded DDZ powder.

### 4.3. Structure Characterization

The hydrolysis degree was determined via the o-phthalaldehyde (OPA) method. L-leucine (0.0820 g) was dissolved and diluted to 25 mL to prepare the standard stock solution, from which a series of standard solutions with gradient concentrations were obtained. OPA working solution (250 mL) was prepared under dark conditions. OPA (0.2 g) was first dissolved in 5 mL of ethanol, followed by the addition of 4.7670 g of sodium tetraborate, 0.25 g of sodium dodecyl sulfate and 0.22 g of dithiothreitol. The mixture was homogenized and diluted to 250 mL. The final concentrations were 0.8 g/L for OPA, 19.068 g/L for sodium tetraborate, 1.0 g/L for sodium dodecyl sulfate and 0.88 g/L for dithiothreitol. The absorbance was measured at 340 nm after a 2 min reaction, and the standard curve was plotted. The absorbance of samples was detected using the same procedure. The degree of hydrolysis was calculated based on the standard curve and the following formula:

HD (%) = [(C_1_ − C_0_)/C_2_] × 100% (1)

Equation:

C_0_ represents the number of amino groups in untreated zein;

C_1_ represents the number of amino groups in zein treated with different concentrations of alkali;

C_2_ represents the number of amino groups in completely hydrolyzed zein (determined by the Kjeldahl method).

The deamidation degree was measured using the phenol–hypochlorite method [28]. Reagent A was prepared by dissolving 5.0 g of phenol and 0.025 g of sodium nitroprusside in deionized water and diluting to 500 mL. Reagent B was obtained by mixing 2.5 g of sodium hydroxide and 4.2 mL of sodium hypochlorite solution and diluting to 500 mL. Both reagents were stored away from light. Ammonium sulfate was dried and formulated into a 10 μg/mL stock solution, which was further diluted to a series of standard solutions (0.2–1.0 μg/mL). After reaction at 37 °C for 20 min, the absorbance was recorded at 625 nm to establish the standard curve. The free ammonia content of samples was determined following the above steps. Total amide content was measured after samples were fully hydrolyzed in 3 mol/L sulfuric acid in boiling water for 3 h and neutralized. The deamidation degree was calculated by the following formula:

DD (%) = [(A_1_ − A_0_)/A_2_] × 100% (2)

Equation:

A_0_ is the absorbance of the amine group in untreated zein;

A_1_ is the absorbance of the amine group in DDZ after treatment with different concentrations of alkali;

A_2_ is the absorbance of the amine group in zein after treatment with 3 mol/L H_2_SO_4_.

Amino acid analysis was performed with slight modifications to the previous method [29]. Zein and DDZ were weighed precisely at 50 mg and subsequently placed into 20.0 mL ampoules. Subsequently, 10.0 mL of 6.0 M hydrochloric acid was added to the ampoules, and the contents were thoroughly mixed. The vacuum-sealed samples were then hydrolyzed at 110 °C in a drying oven for a period of 24 h. The hydrolysate was then subjected to cooling, filtration, and dilution to a final volume of 100.0 mL. Subsequently, 5.0 mL of the solution was evaporated under a nitrogen stream. The dried residue was dissolved in 1.0 mL of sodium citrate buffer (pH 2.2) and injected into a Hitachi L-8900 amino acid analyzer (Tokyo, Japan) for amino acid analysis.

The zeta potential of the samples was measured at 25 °C using a Nano-ZS Zetasizer analyzer (Malvern Instruments, Worcestershire, UK). Prior to measurement, the dispersion was appropriately diluted with deionized water; each sample was measured in triplicate, and the average value was recorded.

The method employed by [28] was utilized with modifications. Zein and DDZ were dissolved in 2% SDS buffer at a concentration of 20.0 mg/mL. The samples were heated for 10 min to ensure complete denaturation, then loaded into the concentrated gel lanes along with the marker. Electrophoresis was initiated at 90 V for 30 min. Once the marker was fully separated, the voltage was increased to 120 V. Finally, the gel was stained with Comas Bright Blue and washed with destaining solution.

FTIR analysis of freeze-dried zein and DDZ samples was performed using a Fourier transform infrared spectrometer (Thermo Fisher, Waltham, MA, USA), and the spectra were analyzed using PeakFit V4.12 software.

### 4.4. Preparation of Q@zein and Q@DDZ

The preparation of 10.0 mg/mL stock solutions of zein and DDZ is to be carried out separately. Subsequently, the pH of each solution was adjusted to 12.0, and the solution was stirred at 600 rpm for 30 min to ensure complete dissolution of DDZ. The carrier protein-to-Q loading ratios were 40:1, 20:1, 10:1, and 5:1, designated as Q1, Q2, Q3, and Q4, respectively. The pH was adjusted to 12.0, and the mixture was stirred for 30 min until complete dissolution was achieved. The pH was then immediately adjusted to 7.0. The resulting mixtures were further stirred at room temperature for 2 h, and then centrifuged at 7155× *g* for 20 min. The supernatant was collected to obtain the corresponding Q@DDZ and Q@zein solutions, which were stored at 4 °C in the dark before further characterization.

### 4.5. Intrinsic Fluorescence Spectroscopy

A protein solution with a mass concentration of 2.0 mg/mL and Q solutions with concentrations of 0.0, 8.0, 16.0, 32.0, 48.0, 64.0, and 80.0 μmol/L were prepared. These solutions were incubated in a water bath at 25 °C for 2 h. After being ice-bathed for 30 min, the samples were removed and allowed to return to room temperature prior to the measurement of their fluorescence emission spectra.

The excitation wavelength was set to 280 nm, and the emission wavelength range was set to 290–500 nm. The scanning speed was set to 1200 nm/min, and both the excitation and emission slit widths were set to 5 nm.

### 4.6. Stern–Volmer Equation Analysis

As previously reported [29], correlation quenching constants were calculated using the Stern–Volmer Equation (3). For static quenching, the binding constant (K_a_) and the number of binding sites (n) were calculated using the double-logarithmic Equation (4).

F_0_/F = 1 + K_sv_ × [Q] = 1 + K_q_ × τ_0_ × [Q] (3)

Equation:

F_0_ and F represent the maximum fluorescence intensities of the sample with and without the addition of Q, respectively;

[Q] is the Q concentration (mol/L);

K_SV_ is the dynamic quenching constant (L/mol);

K_q_ is the molecular quenching rate constant (L/mol·s);

τ_0_ is the average lifetime of a fluorescent molecule in the absence of quenchers, with a magnitude of 10^−8^ s.

lg [(F_0_ − F)/F] = lgK_a_ + *n*lg[Q] (4)

Equation:

K_a_ is the combination constant;

*n* is the binding site.

### 4.7. Fourier Transform Infrared Spectroscopy (FTIR)

Freeze-dried samples were characterized by ATR-FTIR spectroscopy. Powders were pressed tightly on the crystal, and spectra were collected by a Nicolet iN10 spectrometer (Thermo Fisher, USA) within 4000–400 cm^−1^ at 4 cm^−1^ resolution. The amide I band (1600–1700 cm^−1^) was deconvoluted using PeakFit V4.12 software. The baseline was corrected, and the amide I region was fitted with Gaussian peaks corresponding to different secondary structures: β-sheet (1615–1637 cm^−1^ and 1682–1700 cm^−1^), random coil (1638–1650 cm^−1^), α-helix (1651–1660 cm^−1^), and β-turn (1660–1681 cm^−1^). The relative content of each secondary structure component was calculated by integrating the area of the corresponding peaks and dividing by the total area of all fitted peaks in the amide I region.

### 4.8. Particle Size and Zeta-Potential Measurement

The particle size and zeta potential of the Q@zein and Q@DDZ were measured at 25 °C using a Nano-ZS Zetasizer analyzer. Before analysis, the sample dispersion was appropriately diluted with deionized water. Each sample was measured three times, with the average value recorded as the experimental result.

### 4.9. Turbidity

The turbidity of Q@zein and Q@DDZ was measured using a UV–visible spectrophotometer (Hitachi, Tokyo, Japan) at a wavelength of 550 nm. The turbidity of Q@zein and Q@DDZ was expressed as 100-T%, where T represents the transmittance of the sample.

### 4.10. Transmission Electron Microscopy (TEM)

The morphology of Q@zein and Q@DDZ was analyzed using a TEM (JEM-2100, JEOL, Tokyo, Japan) operated at an accelerating voltage of 200 kV. Freshly prepared nanoparticle dispersions were diluted with distilled water. A drop of the diluted dispersion was placed on a 200-mesh carbon-coated copper grid and dried in air. Sample images were taken at different magnifications.

### 4.11. Determination of Surface Hydrophobicity (H_0_)

Using ANS as the fluorescent probe, the concentrations of the Q@zein and Q@DDZ were adjusted to 0.1, 0.2, 0.3, and 0.5 mg/mL. A volume of 4.0 mL from each sample was mixed with 20.0 µL of 8.0 mmol/L ANS solution at pH 7.0. The mixture was analyzed using a fluorescence spectrophotometer (Hitachi, Tokyo, Japan) with excitation and emission wavelengths of 390 and 470 nm, respectively, and a slit width of 5 nm. A linear relationship plot was constructed, with nanoparticle concentration on the x-axis and fluorescence intensity on the y-axis. The slope of the linear equation represents the surface hydrophobicity coefficient (H_0_).

### 4.12. Encapsulation Efficiency (EE) and Loading Capacity (LC)

Q standard samples were dissolved in anhydrous ethanol to prepare a stock solution of quercetin at a concentration of 1 mg/mL. This stock solution was then diluted with anhydrous ethanol to obtain quercetin solutions at concentrations of 2 μg/mL, 4 μg/mL, 6 μg/mL, 8 μg/mL, 10 μg/mL, and 12 μg/mL. The absorbance of quercetin was measured at 374 nm, yielding a standard curve of y = 0.0735x − 0.0378, with R^2^ = 0.9989.

The nanoparticle dispersion in an 80% (*v*/*v*) ethanol solution was diluted. The absorbance of the sample was measured at 374 nm using a UV spectrophotometer. The quercetin concentration was calculated based on the previously determined standard curve, and then the encapsulation efficiency (EE) and drug loading (LC) were calculated using the following formulas:

EE = mass of Q loaded in nanoparticles/quantity of Q added × 100%(5)

LC = mass of Q loaded in nanoparticles/total mass of nanoparticles × 100%(6)

### 4.13. Stability Analysis

#### 4.13.1. pH Stability

Freshly prepared samples were adjusted to different pH values (2.0, 3.0, 4.0, 5.0, 6.0, 7.0, 8.0, and 9.0) using 1.0 M HCl or NaOH. The particle size and zeta potential of the samples under different pH conditions were measured.

#### 4.13.2. Ionic Strength Stability

Freshly prepared samples were mixed with an equal volume of NaCl solution to prepare samples with different NaCl concentrations (0.0, 50.0, 100.0, 150.0, 200.0, and 300.0 mM). The particle size and zeta potential of the nanoparticle dispersion were measured.

#### 4.13.3. Thermal Stability

Freshly prepared samples were placed in a constant temperature water bath at 80 °C for 0–180 min and then immediately cooled to room temperature. The retention rate of Q was determined.

### 4.14. In Vitro Antioxidant Activity Analysis

#### 4.14.1. ABTS Radical Scavenging Activity

The ABTS radical scavenging rate was determined by the reported method [30] with minor modifications. ABTS powder (38.4 mg, 7.0 mmol/L) was dissolved in distilled water and diluted to 10.0 mL. Potassium persulfate powder (10.0 mg, 2.5 mmol/L) was dissolved in distilled water and diluted to 15.0 mL. The two solutions were mixed at a 1:1 volume ratio, thoroughly shaken, and incubated in the dark at room temperature for 12–16 h to prepare the ABTS stock solution. Then, 200.0 μL of the sample solution was mixed with 600.0 μL of the diluted ABTS solution, vortexed, and incubated at room temperature in the dark for 30 min, and the absorbance was measured at 734 nm (denoted as A_1_). For the control group, 200.0 μL of distilled water was used to replace the sample solution; the absorbance was denoted as A_2_. The ABTS radical scavenging rate was calculated using the following formula:

ABTS scavenging rate (%) = (A_2_ − A_1_)/A_2_ × 100% (7)

#### 4.14.2. DPPH Radical Scavenging Activity

The antioxidant activity was determined using the DPPH radical assay according to the previously reported method [31] with slight modifications. A total of 2.0 mL of the sample solution (5.0 mg/mL) was mixed with 2.0 mL of 1.0 mmol/L DPPH ethanol solution, and the mixture was incubated in the dark for 30 min. After the reaction, the absorbance of the mixture was measured at 517 nm (recorded as A_i_). Similarly, the absorbance of the sample mixed with an equal volume of anhydrous ethanol was determined under the same conditions (recorded as A_j_), and the absorbance of anhydrous ethanol mixed with 1.0 mmol/L DPPH ethanol solution was determined as the blank control (recorded as A_c_). The DPPH radical scavenging rate was calculated using the following formula:

DPPH scavenging rate (%) = [1 − (A_i_ − A_j_)/A_c_] × 100% (8)

### 4.15. In Vitro Digestion

Simulated Saliva Fluid (SSF) was prepared (0.2 mg/mL mucin, 1.6 mg/mL NaCl, and 0.2 mg/mL KCl) and mixed with the sample at a 1:1 mass ratio. The mixture was adjusted to pH 6.8 and incubated at 37 °C and 90 rpm for 10 min to simulate oral conditions. The SSF was mixed with Simulated Gastric Fluid (SGF; 2.0 mg/mL NaCl and 3.2 mg/mL pepsin, pH 2.0) at a 1:1 (*v*:*v*) ratio, and digested at 37 °C and 200 rpm for 2.0 h. The pH was neutralized immediately. Then, an equal volume of Simulated Intestinal Fluid (SIF; pH 7.2, 10.0 mg/mL bile salts, and 1.5 mg/mL trypsin) was added for digestion for 2.0 h. The release rate of soluble Q was determined at different time points (0, 10, 30, 60, 90, 120, 150, 180, 210, 240, 270, and 300 min). The Q release rate was analyzed as described in Section 4.12.

### 4.16. Statistical Analysis

All data were the average values of triplicate determinations. Excel was used for data calculation, and Origin was used for graphing. The results were analyzed by one-way analysis of variance (ANOVA) using SPSS 25.0 software.

## Figures and Tables

**Table 1:** Hydrolytic degree and deamidation of DDZ.

Sample	Degree of Hydrolysis (%)	Degree of Deamidation (%)
DDZ	5.95 ± 0.05	8.51 ± 0.27

**Table 2:** Binding constants and binding sites of zein and DDZ with Q.

Protein	K_q_ (10^12^ L/mol·s)	K_a_ (10^3^ L/mol)	*n*
zein	4.70	1.61	0.8884
DDZ	1.36	2.25	1.7561

## Data Availability

The original contributions presented in the study are included in the article; further inquiries can be directed to the corresponding author.

## Abbreviations

The following abbreviations are used in this manuscript:

Q	Quercetin
DDZ	Deamidated zein peptide
Gln	Glutamine
Asn	Asparagine
Asp	Aspartic
Glu	Glutamic
Tyr	Tyrosine
Leu	Leucine
Ile	Isoleucine
Phe	Phenylalanine
FTIR	Fourier transform infrared spectroscopy
TEM	Transmission electron microscopy
EE	Encapsulation efficiency
LC	Loading capacity
pI	Isoelectric point
